# TRK Inhibitors in Adult and Pediatric High-Grade Gliomas: A Systematic Review and Individual Participant Data Meta-Analysis

**DOI:** 10.3390/cancers17132089

**Published:** 2025-06-23

**Authors:** Massimiliano Domenico Rizzaro, Claudia Fanizzi, Giorgio Fiore, Luigi Gianmaria Remore, Antonella Maria Ampollini, Mauro Pluderi, Manuela Caroli, Marco Locatelli

**Affiliations:** 1 Neurosurgery Unit, Fondazione IRCCS Ca’ Granda Ospedale Maggiore Policlinico, 20122 Milan, Italy; claudia.fanizzi@policlinico.mi.it (C.F.); giorgio.fiore@policlinico.mi.it (G.F.); luigi.remore@policlinico.mi.it (L.G.R.); antonella.ampollini@policlinico.mi.it (A.M.A.); mauro.pluderi@policlinico.mi.it (M.P.); manuela.caroli@policlinico.mi.it (M.C.); marco.locatelli@unimi.it (M.L.); 2 Department of Pathophysiology and Transplantation, University of Milan, 20122 Milan, Italy

**Keywords:** high grade glioma, TRK inhibitor, larotrectinib, pediatric, NTRK gene fusion

## Abstract

Background: High-grade glioma (HGG) is the most common primary malignant brain tumor, with peak incidence in the fifth and sixth decades of life. Although HGG is rare in children, the prognosis remains poor, with a median overall survival (OS) of less than two years. Recently, TRK inhibitors have been approved for the treatment of tumors harboring NTRK gene fusions. In this review, we analyzed data from early clinical trials investigating the use of these agents in patients with HGG. Methods: A systematic literature search was performed in the PubMed database. Studies involving patients with HGG treated with TRK inhibitors were included. We analyzed progression-free survival (PFS), 24-week disease control rate, and complete or partial radiological responses according to the Response Assessment in Neuro-Oncology (RANO) criteria. Results: Sixteen studies comprising 55 patients with HGG harboring NTRK gene fusions (19 adults and 36 children) were included. A statistically significant difference in PFS was observed between pediatric and adult patients treated with TRK inhibitors (17 vs. 8.5 months; *p* < 0.001). Pediatric patients also exhibited a higher rate of complete or partial radiological response compared to adults (94% vs. 57%). Discussion: Although the available evidence on TRK inhibitors in HGG is limited, the findings of this review highlight a potentially promising role for these agents, particularly in the treatment of pediatric HGGs.

## Simple Summary

This review investigates the role of a novel class of drugs—tropomyosin receptor kinase (TRK) inhibitors—used in solid tumors characterized by neurotrophic tyrosine receptor kinase (NTRK) gene fusions. We selected 16 studies in which TRK inhibitors were administered as adjuvant therapy in patients with high-grade gliomas. Survival outcomes and treatment responses, assessed according to the Response Assessment in Neuro-Oncology (RANO) criteria, were reported and compared between pediatric and adult patient groups. Our findings indicate that the use of TRK inhibitors is associated with higher progression-free survival (PFS) and a greater RANO response rate in pediatric patients compared to adults.

## 1. Introduction

High-grade gliomas (HGGs) are the most prevalent primary malignant brain tumors, with an incidence of approximately 5 per 100,000 person-years in Europe and North America [1]. Although these tumors can occur across all age groups, their incidence peaks during the fifth and sixth decades of life [2,3]. Pediatric high-grade gliomas (pHGGs), however, represent a distinct clinical and biological entity from their adult counterparts (aHGGs). According to the latest World Health Organization (WHO) classification, pHGGs are categorized into four molecular subtypes: (1) H3 K27-altered diffuse midline gliomas, (2) H3 G34-mutant diffuse hemispheric gliomas, (3) H3-wildtype and IDH-wildtype diffuse pediatric-type high-grade gliomas, and (4) infant-type hemispheric gliomas [4].

Spinal HGGs are rare, accounting for only 1.5% of all spinal cord tumors and approximately 7.5% of all intramedullary gliomas [5]. The current standard of care for aHGGs involves maximal safe surgical resection followed by adjuvant chemoradiotherapy [6], a regimen that has seen limited innovation since the introduction of the Stupp protocol [7].

The prognosis for pHGGs remains particularly poor, especially in children under the age of three, due to reduced responsiveness to temozolomide (TMZ) and heightened vulnerability to the adverse effects of radiotherapy [8]. This is compounded by the anatomical location of the most common pHGG subtype—diffuse midline gliomas—which arise in deep-seated brain structures and are typically not amenable to radical surgical excision [8]. Although various second-line therapies have been explored, none have demonstrated substantial clinical benefit to date [9,10,11,12].

The average survival of an adult patient with high-grade glioma ranges from 13 to 24 months, while in pediatric cases, it can vary from 10 to 75 months [2]. The median survival of pediatric patients is less well characterized in the literature. This is due to the rarity of these tumors and recent changes in their nosological classifications, which have limited the chances to aggregate more comprehensive data on this tumor type.

In recent years, numerous genetic alterations have been identified in high-grade gliomas (HGGs), with key driver mutations involving IDH, EGFR, TERT, PTEN, TP53, ALK, and ROS1, which serve as critical hub proteins in glioma tumorigenesis [13,14]. A significant advancement in targeted therapy came with the FDA (U.S. Food and Drug Administration) approval of larotrectinib in November 2018 for solid tumors harboring NTRK (neurotrophic tyrosine receptor kinase) gene fusions. This was followed by the approvals of entrectinib in 2019 and repotrectinib in 2024. The *NTRK1*, *NTRK2*, and *NTRK3* genes encode the TRKA, TRKB, and TRKC receptors, respectively—tyrosine kinase receptors that, upon dimerization, activate oncogenic signaling cascades such as the MAPK, PI3K/AKT, and PLC-γ pathways [15].

TRK receptors are physiologically expressed at high levels in neural tissue, where they regulate essential processes including neuronal survival, development, proliferation, synaptic plasticity, and higher cognitive functions such as learning and memory [16,17]. When NTRK genes undergo oncogenic fusion events with unrelated genes, the resulting chimeric proteins can lead to constitutive TRK activation and downstream tumorigenesis [18]. These gene fusions have been detected in approximately 2% of all gliomas [19], and notably, in up to 40% of non-brainstem HGGs occurring in children under 3 years of age [20].

This review aims to evaluate the clinical outcomes of HGG patients harboring NTRK fusion mutations who were treated with TRK inhibitors and to contribute new comparative data regarding survival outcomes between pediatric and adult populations treated with these targeted agents.

## 2. Materials and Methods

### 2.1. Literature Search

**Figure 1:**
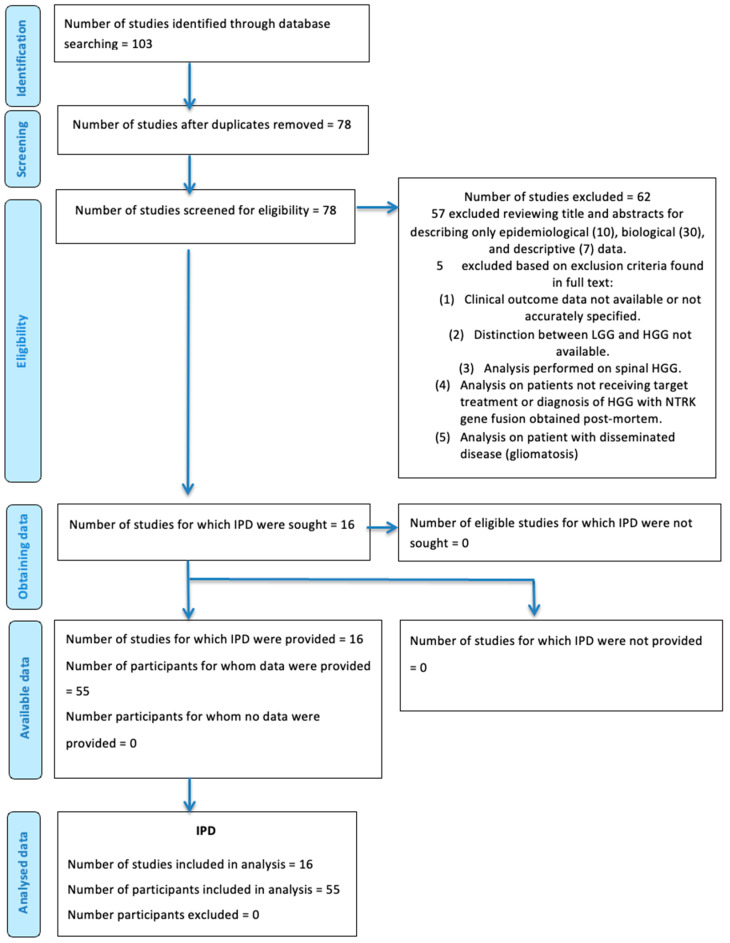
PRISMA flow diagram for individual participant data (IPD) meta-analyses and systematic review.

The literature review was conducted in accordance with the Cochrane Handbook for Systematic Reviews and adhered to the Preferred Reporting Items for Systematic Reviews and Meta-Analyses (PRISMA) guidelines [21]. The PRISMA checklist is available in the Supplementary Materials (Figure S1), and the corresponding PRISMA flow diagram is presented in Figure 1. This review was not preregistered in a systematic review database, and no formal review protocol was developed.

A comprehensive literature search was performed using the PubMed and Google Scholar databases. The primary search strategy included the keywords “TRK inhibitors” AND (“glioma” OR “high-grade glioma” OR “glioblastoma”). An additional search was conducted combining the term “glioma” with specific TRK inhibitor names: “larotrectinib”, “entrectinib”, “repotrectinib”, and “selitrectinib”. The reference lists of all included articles were also manually screened to identify any additional studies that met the inclusion criteria. The final search was completed on 1 December 2024.

### 2.2. Inclusion and Exclusion Criteria

In accordance with the population–intervention–comparator–outcomes (PICO) framework, two independent reviewers (M.D.R. and C.F.) conducted a systematic literature search and defined the eligibility criteria for study inclusion.

Studies were considered eligible if they met the following criteria:

(1) Included at least one patient with a histological and molecular diagnosis of intracranial high-grade glioma (HGG) harboring NTRK gene fusions or mutations.
(2) Reported treatment with TRK inhibitors (either as first-line or adjuvant therapy).
(3) Were published in English.

Exclusion criteria were as follows:

(1) Studies focusing solely on the biological or molecular mechanisms of TRK inhibitors (e.g., in vitro or animal models) without reporting clinical outcomes.
(2) Studies involving only patients with a histological or molecular diagnosis of low-grade glioma (LGG).
(3) Reports on patients receiving TRK inhibitors without a confirmed histological diagnosis (e.g., under compassionate use protocols).
(4) Studies including patients with encephalic or spinal gliomatosis.
(5) Studies including patients with spinal high-grade gliomas.
(6) Studies lacking individual patient-level data or those that did not report clinical outcomes following TRK inhibitor treatment.

Study selection occurred in two phases. Initially, titles and abstracts of retrieved articles were screened for relevance. Subsequently, the full texts of potentially eligible articles were reviewed in detail to confirm inclusion. This selection process was independently performed by both reviewers (M.D.R. and C.F.) on two separate occasions. Any disagreements or uncertainties regarding study eligibility were resolved through consensus with the senior author (M.L.). Reference lists of included studies were also screened to identify any additional relevant publications.

### 2.3. Data Extraction and Quality Assessment

Two independent reviewers (M.D.R. and C.F.) extracted the following data from each included study: sample size, patient demographics, histological and molecular tumor characteristics, treatment details, clinical and radiological outcomes, presence of NTRK1, NTRK2, or NTRK3 gene fusions or mutations, co-occurring genetic alterations, type and dosage of TRK inhibitor administered, timing of TRK inhibitor initiation relative to diagnosis, and tumor volume at follow-up MRI.

Clinical response and outcome measures included progression-free survival (PFS), 24-week disease control rate (from the initiation of TRK inhibitor therapy), and tumor volume reduction. Radiological response was categorized as complete response, partial response, or stable disease, based on the Response Assessment in Neuro-Oncology (RANO) criteria [22].

Individual patient-level data were extracted when explicitly reported in the text, tables, or figures. The level of evidence for each study was assessed according to the 2011 Oxford Centre for Evidence-Based Medicine guidelines [23]. Risk of bias was evaluated using the Joanna Briggs Institute (JBI) critical appraisal tools for case reports, case series, and clinical trials [24,25]. The PRISMA flow diagram was used to illustrate the study selection process [26]. The overall reporting quality of the studies was assessed using relevant CONSORT extensions [27]. Evaluation of level of evidence, bias, and study quality was independently performed by both reviewers (M.D.R. and C.F.).

### 2.4. Statistical Analyses

Descriptive statistics, including frequencies and percentages, were used to summarize demographic characteristics. The Shapiro–Wilk test was applied to assess the normality of continuous variables. Clinical outcomes following TRK inhibitor administration—namely, progression-free survival (PFS), 24-week disease control rate, radiological response according to RANO criteria, PFS among patients achieving 24-week disease control, and tumor volume reduction on follow-up MRI—were compared between pediatric and adult patient groups. Mean differences between these groups were calculated accordingly.

PFS values were directly extracted from the original studies. Patients who were still undergoing treatment or who had not experienced disease progression at the time of publication were considered censored in the Kaplan–Meier (KM) survival analysis. Group comparisons of PFS between pediatric and adult populations were performed using the log-rank test.

Tumor volume reduction was assessed based on the numerical data provided in the studies; no independent volumetric analyses were conducted on the MRI images. A two-tailed t-test was used to compare continuous variables. Statistical significance was defined as a *p*-value < 0.05.

Interstudy heterogeneity was evaluated using the I^2^ statistic with a two-stage approach. To assess the robustness of findings, a sensitivity analysis was conducted using an as-treated model. All statistical analyses were performed using SPSS software (version 27.0; IBM Corp., Armonk, NY, USA).

## 3. Results

### 3.1. Study Selection

The online literature search initially identified 103 studies. After removing 25 duplicates, 78 studies were screened based on title and abstract. Among these, 47 studies were excluded because they focused solely on the epidemiological (*n* = 10), biological (*n* = 30), or descriptive (*n* = 7) aspects of gliomas harboring *NTRK* gene fusions.

Of the 31 remaining studies, 10 were excluded for the following reasons: analysis focused on low-grade gliomas (*n* = 8), sarcomas (*n* = 1), or glioneuronal tumors (*n* = 1). The full texts of the remaining 21 studies were assessed for eligibility. Five were excluded due to non-compliance with inclusion criteria, which included (1) absence or inaccuracy of clinical outcome data, (2) lack of distinction between low-grade and high-grade gliomas, (3) focus on spinal high-grade gliomas, (4) patients either not treated with targeted therapies or diagnosed post-mortem, and (5) inclusion of disseminated disease (gliomatosis cerebri).

Ultimately, 16 studies met all eligibility criteria and were included in this review. These comprised two clinical trials (NCT02637687, NCT02576431) [28], one case series [29], six case reports [30,31,32,33,34,35] and one retrospective cohort study [36]. An additional six case reports [37,38,39,40,41,42] were included following a supplementary search using the specific names of the drugs (see Figure 1). Three of these reports were technically case series; however, only one patient per series was eligible for inclusion based on our predefined criteria.

The inter-reviewer agreement for study selection exceeded 90%, and the included studies demonstrated a low risk of bias as assessed by the Joanna Briggs Institute (JBI) critical appraisal tools (mean risk of bias: 11.6%). Individual patient data (IPD) were available for all 55 patients and were extracted either directly from the text or reconstructed from figures and tables. This enabled an IPD meta-analysis.

The two-stage I^2^ statistic yielded a value of 10%, indicating low heterogeneity among included studies. A sensitivity analysis using an as-treated approach confirmed the robustness of the findings. Given that outcome data were consistently extracted across all studies, the risk of bias due to missing data was considered negligible despite the high number of case reports. The selected studies reported survival data on an individual patient level, allowing us to use it as the primary outcome of this review.

### 3.2. Demographic Data

A total of 55 patients were included in the analysis, comprising 36 pediatric and 19 adult cases. The mean age in the pediatric cohort was 2.9 years (SD ± 3 years), while the mean age among adults was 52.4 years (SD ± 8 years). In the pediatric group, 62% were female, whereas in the adult group, 59% were male. All gliomas included were classified as high-grade tumors, based on histopathological and molecular diagnostic criteria. The vast majority of tumors (54 out of 55) were supratentorial, with only one located in the cerebellum. Molecular profiling revealed that 72% of gliomas harbored NTRK2 fusions, while NTRK1 and NTRK3 fusions were identified in 13% and 15% of cases, respectively. In the pediatric cohort, 89% of patients underwent surgical resection, whereas the remaining underwent biopsy for diagnostic confirmation. Among adults, gross total resection was performed in 93% of cases. Adjuvant chemotherapy was administered in 92% of pediatric and 96% of adult patients. The most commonly used chemotherapeutic agent was temozolomide (TMZ), followed by lomustine, vincristine, and procarbazine. One pediatric case received neoadjuvant chemotherapy in combination with stem cell transplantation. Adjuvant radiotherapy was delivered to 74% of pediatric and 85% of adult patients. Overall, more than half of patients in both cohorts underwent multiple cycles of radio-chemotherapy prior to receiving TRK inhibitor therapy. The most frequently used TRK inhibitor was larotrectinib, with an average dosage of 100 mg/m^2^ twice daily. Entrectinib and repotrectinib were each used in one case. On average, TRK inhibitors were introduced 12.8 months after diagnosis in the pediatric group and 14.5 months in the adult group. Notably, larotrectinib was used as first-line therapy in only one pediatric patient. A summary of the key findings from the included studies is provided in Table 1.

### 3.3. Clinical Outcome and Comparison Between the Two Groups

The median progression-free survival (PFS) for pediatric patients was 17 months following the initiation of TRK inhibitor therapy. The 24-week disease control rate (DCR) in this group was approximately 88%. Notably, 55% and 31% of pediatric patients maintained at least stable disease (SD) for 1 year and 2 years, respectively, after starting treatment. Radiological response—defined as at least a partial response per RANO criteria for measurable disease—was observed in 94% of pediatric patients, with a mean reduction in residual tumor volume of 52%.

In contrast, the adult cohort had a mean PFS of 8.5 months after TRK inhibitor initiation. The 24-week DCR was 83%. Only 26% and 5% of adults demonstrated disease stability for 1 year and 2 years, respectively. All adult patients either experienced disease progression or died within two years of starting TRK inhibitor therapy. Radiologic response was observed in 57% of adult patients on follow-up MRI, while 29% showed radiologic disease progression on the first post-treatment scan, compared to 6% of pediatric cases.

**Figure 2:**
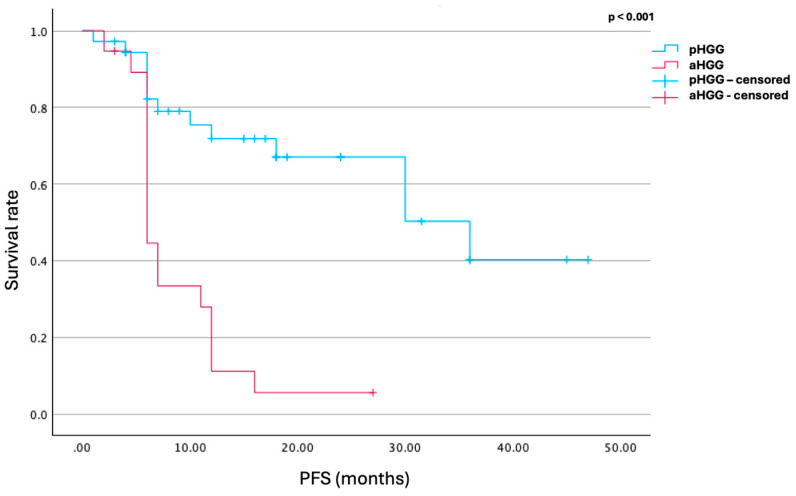
Kaplan–Meier curve illustrating progression-free survival (PFS) following TRK inhibitor therapy in pediatric (pHGG) and adult (aHGG) high-grade glioma patients. The mean PFS was 17 months in the pediatric cohort and 8.5 months in the adult cohort (*p* < 0.001), indicating significantly longer disease control in children. red: aHGG, blue: pHGG.

Overall, these data suggest that TRK inhibitors are associated with significantly improved PFS in pediatric patients with high-grade gliomas compared to adults (17 ± 10.5 vs. 8.5 ± 3.5 months, *p* < 0.001). Although the 24-week DCR was similar between groups (88% in pediatric vs. 83% in adults), pediatric patients with disease control at 24 weeks had a notably longer mean PFS (18.6 ± 10 months vs. 8 ± 3 months, *p* = 0.005). Additionally, complete or partial responses were more frequent in the pediatric group (94% vs. 57%) based on RANO criteria. The mean tumor volume reduction was significantly greater in children (0.52 ± 0.4 vs. 0.07 ± 0.36, *p* = 0.018) (Figure 2, Table 2).

Furthermore, the use of TRK inhibitors was associated with an improvement in overall survival (OS) compared to historical averages reported in the literature: 22 vs. 14 months for adult HGGs and 30 vs. 20 months for pediatric HGGs, respectively.

Regarding safety, treatment-related adverse events were mostly Grade 1–2, as per the Common Terminology Criteria for Adverse Events (CTCAE). Grade 3–4 events occurred in 18% of patients, most commonly including neutropenia (7%), elevated alanine aminotransferase (3%), and elevated aspartate aminotransferase (2%).

## 4. Discussion

With the advancement of molecular profiling technologies, there has been a growing trend toward treating cancer with therapies targeting specific genetic alterations, consistent with the principles of precision medicine [43]. The recently published INDIGO trial demonstrated that vorasidenib, an inhibitor of mutant isocitrate dehydrogenase 1 and 2 (IDH1/2), significantly improved progression-free survival compared to placebo in patients with low-grade IDH-mutant gliomas [44]. However, in high-grade gliomas (HGGs), where multiple genetic and epigenetic alterations are often present and poorly understood, the application of targeted therapies has remained limited. The most promising outcomes thus far have involved anti-vascular endothelial growth factor agents, such as bevacizumab and, more recently, regorafenib, though neither has significantly altered the poor prognosis of HGGs [45,46,47,48]. This highlights the urgent need to further investigate molecular abnormalities in HGGs to identify new therapeutic targets that can improve patient survival. In 2018, the global incidence of solid tumors harboring NTRK fusions was estimated at 0.52 per 100,000 persons [49]. These genetic alterations are far more prevalent in certain rare tumors, such as infantile fibrosarcoma (90.6%), secretory breast carcinoma (92.9%), secretory salivary gland carcinoma (79.7%), and congenital mesoblastic nephroma (21.5%) [50]. Recent studies profiling various solid tumors found that concurrent oncogenic alterations in genes such as ALK, BRAF, ERBB2, EGFR, ROS1, and KRAS are uncommon in tumors with NTRK fusions. This supports the hypothesis that NTRK fusions act as primary oncogenic drivers, reinforcing the importance of identifying patients with TRK fusion-positive cancers [51,52,53].

The high genetic complexity of adult high-grade gliomas (aHGGs) poses a major challenge to the development of effective targeted therapies. The presence of multiple oncogenic “driver” mutations may render targeted therapy less effective, as tumor cells can bypass inhibition by activating alternative oncogenic pathways [54]. Melanoma exemplifies this challenge, where combination therapies targeting multiple pathways have already been integrated into clinical practice [55]. In HGGs, resistance mechanisms to drugs such as bevacizumab—including proangiogenic and proinvasive responses—have also been described [56]. Conversely, pediatric high-grade gliomas (pHGGs), which often exhibit fewer mutations, may be more amenable to targeted therapy. A notable example is infant-type hemispheric glioma, which tends to have a better prognosis due to well-defined and targetable molecular alterations. In such cases, the presence of single mutations (e.g., ALK, ROS, NTRK) allows for the use of highly specific therapies that may improve outcomes. Typically, NTRK fusion events involve the NTRK1 or NTRK3 genes, whereas NTRK2 mutations are more frequently observed in primary brain tumors [57]. Although numerous reviews have examined the efficacy of TRK inhibitors in solid tumors, few have specifically focused on central nervous system (CNS) tumors. Wang et al. published two reviews discussing TRK inhibitors in solid and CNS tumors but did not emphasize HGGs [58,59], while Lang et al. exclusively focused on pediatric CNS tumors [60]. More recently, Lamoureux et al. conducted a retrospective study involving 119 patients from 49 centers with CNS tumors harboring confirmed TRK fusions. However, only 57.1% of the included cases were HGGs, and the study did not report the tumor grade or therapeutic approaches specifically for Grade 3 and 4 gliomas, limiting interpretability [36]. Furthermore, the heterogeneity in histological types and prior treatments across the cohort complicates the extraction of definitive conclusions. NTRK fusions have been reported in approximately 2% of adult HGGs and up to 6.2% of pediatric cases [36,61]. However, this may underestimate the true prevalence in adults, where NTRK testing is not routinely performed. In contrast, molecular testing is more commonly performed in pediatric brain tumors [62]. Pediatric patients may also derive greater benefit from targeted therapies due to the limitations of conventional treatments—such as chemotherapy and radiotherapy—which can cause significant hematologic and neurologic toxicity. Targeted therapies, including TRK inhibitors, typically exhibit a more favorable safety profile [63,64,65,66,67].

A key limitation of the available literature is the inconsistent reporting of survival outcomes alongside molecular data, which impedes the ability to attribute prognostic significance to individual mutations. Only case reports, which comprised a small portion of our dataset, provided detailed molecular characterizations. Including these limited cases in our broader analysis would have introduced significant bias and reduced overall reliability. Our review suggests that TRK inhibitors may be more effective in pHGGs than aHGGs, as evidenced by significantly longer progression-free survival (PFS) in the pediatric cohort. However, it is important to acknowledge potential detection and information bias, particularly since NTRK fusions are infrequently tested in aHGGs. Additionally, while pathogenetically related, pHGGs and aHGGs are considered distinct disease entities with markedly different genetic profiles, treatment approaches, and prognoses. Therefore, comparing treatment outcomes between these groups has inherent limitations.

Furthermore, the pHGG cohort encompasses various tumor subtypes with diverse prognoses. Among these, infant-type hemispheric glioma demonstrates a notably favorable outcome due to its distinct molecular profile and responsiveness to targeted therapies [68]. Specifically, patients with ALK rearrangements had superior 5-year overall survival rates compared to those with ROS1 alterations (53.8% vs. 25%), while those with NTRK fusions showed intermediate outcomes [68]. The inclusion of such cases in our analysis may have influenced overall findings and reduced their generalizability, particularly since individual histological diagnoses were not consistently reported across studies. Future investigations should aim to correlate PFS with specific tumor subtypes and molecular alterations to refine prognostic assessments.

Since their introduction in glioma therapy, TRK inhibitors have been primarily administered in combination with other treatments. The cohorts analyzed in this review were characterized by substantial heterogeneity in terms of clinical features, treatment history, and histopathology. The total number of patients included (*n* = 55) limits the statistical power to definitively assess the impact of TRK inhibitors on survival outcomes. Notably, TRK inhibitors were frequently administered as salvage therapy in heavily pretreated patients—especially in aHGGs—often after three or more prior treatment lines (see Table 2). As a result, the observed effects may have been diminished by treatment delays and disease refractoriness. While our analysis is based on a highly heterogeneous cohort, the consistent use of TRK inhibitors as a last-line option may serve as a natural form of standardization by focusing on treatment-refractory populations. Despite differences in disease biology, comparing pHGG and aHGG outcomes remains relevant given their similar standard treatment protocols and the consistent timing of TRK inhibitor use (post-standard therapy failure). Based on the studies included, TRK inhibitors were associated with modest survival benefits in both groups when considered separately, with a more pronounced effect in pediatric patients. However, patients with longer survival may inherently represent a subgroup with more favorable tumor biology, potentially skewing results. One of the key findings of our review is the lack of robust data regarding the comprehensive molecular profile of HGGs, which limits our ability to draw definitive conclusions. Moreover, the small sample size and lack of uniform reporting across studies represent significant constraints. Nevertheless, the availability of individual patient-level data on PFS and radiologic response enabled us to perform an individual patient data (IPD) meta-analysis, allowing for more granular statistical evaluation and enhancing the reliability of our findings despite inherent limitations.

## 5. Conclusions

TRK inhibitors represent a promising therapeutic option for high-grade gliomas (HGGs), particularly in pediatric populations. Due to the limited availability of data, our review was primarily based on small case series. Despite these limitations, the observed improvement in progression-free survival (PFS) among pediatric HGG (pHGG) patients suggests that NTRK inhibitors warrant further investigation in adults. To fully elucidate the therapeutic potential of these agents, prospective, randomized studies with larger and more homogeneous cohorts are necessary. Additionally, future research should aim to determine the optimal timing for the introduction of TRK inhibitors within the disease course to maximize clinical benefit.

## Figures and Tables

**Table 1:** Demographic and clinical data. *: mean value; A: adult; P: pediatric; JBI: Joanna Briggs Institute.

Study	Sample	Age (y/o)	Therapeutic Lines Before TRK Inhibitor (Patients)	PFS from Drug Introduction (Months)	Risk of Bias (JBI)
Doz et al. [28]	A: 6	50 *	3 (2) 4 or more (4)	7 *	Low (8%)
	P: 12	6 *	1 (1) 2 (4) 3 (2) 4 or more (5)	12 *	
Kim et al. [29]	A: 1	67	3	6	Low (11%)
	P: 1	2	3	36	
Ziegler et al. [30]	P: 1	3	3	9	Low (25%)
Simoneau et al. [31]	P: 1	11 months	0	36	Low (12%)
Alharbi et al. [32]	P: 1	18 months	1	8	Low (12%)
Mangum et al. [33]	P: 1	6	2	10	Low (12%)
Waters et al. [34]	P: 1	8 months	2	12	Low (12%)
Barritault et al. [35]	P: 1	1	4	8	Low (12%)
Lamoureux et al. [36]	A: 11 P: 12	50 * 4.5 *	3 (4) 4 or more (7) 1 (7) 2 (1) 3 (1) 4 or more (3)	8 * 19.5 *	Low (8%)
König et al. [37]	A: 1	80	1	3	Low (0%)
Mançano et al. [38]	P: 1	9 months	2	8	Low (25%)
Carter-Febres et al. [39]	P: 1	/	2	15	Low (25%)
Pearce et al. [40]	P: 1	<5	2	45	Low (12%)
Di Ruscio et al. [41]	P: 1	<1	3	47	Low (0%)
Keddy et al. [42]	P: 1	3	2	6	Low (0%)

**Table 2:** Table showing highest survival and response rate to TRK inhibitors in children (pHGG) compared with adults (aHGG).

	aHGG	pHGG	*p*-Value
**Mean PFS from drug introduction (months)**	8.5 (±3.5)	17 (±10.5)	<0.001
**Mean tumor volume reduction from drug introduction**	0.07 (±0.36)	0.52 (±0.4)	0.018
**Mean PFS for patients with 24-week disease control (months)**	8 (±3)	18.6 (±10)	0.005
**Twenty-four-week disease control rate**	83%	88%	
**Patients with complete or partial response at first MRI follow-up (RANO)**	57%	94%	

## Supplementary Materials

The following supporting information can be downloaded at: https://www.mdpi.com/article/10.3390/cancers17132089/s1, Figure S1: PRISMA statement or flow diagram.

## Abbreviations

NTRK	neurotrophic tyrosine receptor kinase
OS	overall survival
PFS	progression-free survival
aHGG	adult high-grade glioma
pHGG	pediatric high-grade glioma
RANO	Response Assessment in Neuro-Oncology
